# Human cytomegalovirus pp65 peptide-induced autoantibodies cross-reacts with TAF9 protein and induces lupus-like autoimmunity in BALB/c mice

**DOI:** 10.1038/s41598-020-66804-1

**Published:** 2020-06-15

**Authors:** Ao-Ho Hsieh, Chang-Fu Kuo, I-Jun Chou, Wen-Yi Tseng, Yen-Fu Chen, Kuang-Hui Yu, Shue-Fen Luo

**Affiliations:** 1 Division of Rheumatology, Allergy and Immunology, Chang Gung Memorial Hospital, Taoyuan, Taiwan; 2 Center for Artificial Intelligence in Medicine, Chang Gung Memorial Hospital, Taoyuan, Taiwan; 3 School of Medicine, Chang Gung University, Taoyuan, Taiwan; 4 Division of Clinical Neurology, School of Medicine, University of Nottingham, Nottingham, UK; 5 Division of Paediatric Neurology, Chang Gung Memorial Hospital, Taoyuan, Taiwan; 6 Division of Rheumatology, Allergy and Immunology, Chang Gung Memorial Hospital, Keelung, Taiwan; 7 Kennedy Institute, University of Oxford, Oxford, UK

**Keywords:** Systemic lupus erythematosus, Autoimmunity

## Abstract

Human cytomegalovirus (HCMV) has been linked to the triggering of systemic lupus erythematosus (SLE). We proposed that B cell epitope region of HCMV phosphoprotein 65 (HCMVpp65)_422–439_ mimics an endogenous antigen and initiates lupus-like autoimmunity. Amino acid homology between HCMVpp65_422-439_ and TAF9_134-144_ (TATA-box binding protein associated factor 9, TAF9) was investigated using a similarity search in NCBI protein BLAST program (BLASTP). A murine model was used to confirm their antigenicity and ability to induce lupus-like symptoms. HCMVpp65_422-439_ induced immune responses with the presence of specific antibodies against HCMVpp65_422-439_ and TAF9_134-144_, as well as anti-nuclear and anti-double-stranded (ds)DNA antibodies that are characteristic of SLE. In addition, the majority of HCMVpp65_422-439_ and TAF9_134-144_ immunized mice developed proteinuria, and their renal pathology revealed glomerulonephritis with typical abnormalities, such as mesangial hypercellularity and immune complex deposition. Immunoglobulin eluted from the glomeruli of HCMVpp65_422-439_ immunized mice showed cross-reactivity with TAF9_134-144_ and dsDNA. Increased anti-TAF9 antibody activity was also observed in the sera from SLE patients compared with healthy people and disease controls. Molecular mimicry between HCMVpp65 peptide and host protein has the potential to drive lupus-like autoimmunity. This proof-of-concept study highlights the mechanisms underlying the link between HCMV infection and the induction of SLE.

## Introduction

Systemic lupus erythematosus (SLE) is an idiopathic autoimmune disease characterized by the production of various antibodies against self-antigens, and tissue inflammatory responses that lead to severe organ damage. Infection and the subsequent immune response can result in persistent autoimmunity in genetically predisposed individuals. Human cytomegalovirus (HCMV) is a common pathogen, which is frequently seen in SLE and evokes disease via autoimmune like components^1^. A possible mechanism that HCMV proteins share homologous sequence with the host and the cross-reactivity between viral and the host protein potentially contributes to the autoimmune response^2,3^. The increased anti-HCMV IgM/IgG titer is often accompanied by the clinical and immunological presentation of SLE and has therefore been considered as a potential pathogenic agent in SLE^4,5^.

Mouse model studies offer an understanding concerning the role of CMV in SLE. NZB/W F1 mouse, an F1 hybrid of New Zealand black female and New Zealand white male, and MRL/MpJ mouse with mutation of Fas gene (MRL/MpJ-Fas^lpr^) are spontaneous models for SLE. The Th1-prone C57BL/6 and Th2-prone BALB/c are commonly used mouse strain for genetic and pathogenic investigation. Animal studies reported that CMV infection is linked with the development of SLE. For example, sialic acid-binding Ig-type lectin H (Siglec-H), a DAP12-associated receptor on pDCs, modulates the secretion of IFN-α in plasmacytoid dendritic cells (pDCs)^6,7^. The elevated level of IFN-α in Siglec-H knockout (KO) mice did not promote the virus clearance after murine CMV (MCMV) infection but instead caused more severe lupus-like symptoms^8,9^. It has been demonstrated that MCMV neutralizing monoclonal antibody (mAb) cross-reacted with MCMV structural proteins and human nuclear U1 small nuclear ribonucleoprotein (U1 snRNP) autoantigen^10^. The administration of mice by this mAb led to edema of the hypodermis and mesangial proliferation in glomeruli^10^. Moreover, the M83 open reading frame (ORF) of MCMV is homologs to HCMV phosphoprotein 65 (HCMVpp65). Direct immunization of NZB/W F1 mice with plasmid DNA encoding MCMV M83 or HCMVpp65 proteins elicited early production of anti-dsDNA antibody and resulted in severe glomerulonephritis^11^.

HCMV phosphoprotein 65 (HCMVpp65) is a tegument protein found in abundance in the virion; it suppresses the host’s immune response by inhibiting expression of the host’s class II major histocompatibility (MHC) and attenuating interferon responses^12–14^. In healthy hosts, dominant T cell epitopes in HCMVpp65 elicit vigorous and specific cytotoxic T lymphocyte activity, whereas SLE patients tend to have a significantly higher prevalence of IgG antibodies against the HCMVpp65 protein compared with normal or other disease controls^11,15^. The B cell epitopes of HCMVpp65 (HCMVpp65_336-561_) have been fine-mapped to the hotspot region of HCMVpp65_422-439_ in our previous works^16,17^. The immunization of mice with either HCMVpp65_336-439_ or HCMVpp65_422-439,_ together with murine C3d, has been shown to induce lupus-like autoantibodies and the subsequent development of autoimmunity^16,17^. At present, HCMVpp65_428-437-GASTSAGRKR_ is thought to be a critical epitope for provoking autoantibody production^17^.

The link between HCMVpp65 and the induction of SLE has rarely been investigated. A previous study by the authors revealed that animals who received the HCMVpp65_422-439_ epitope produced hallmark serological and renal features, which resembled human SLE^17^. The authors postulated that cross-reactive antibodies produced in TATA-box binding protein associated factor 9 (TAF9)_134–144_ or HCMVpp65_422-439_ immunized mice may lead to epitope spreading and contribute to the pathogenesis of glomerulonephritis. To verify this hypothesis, a murine model was designed, which immunized mice with a peptide-C3d complex with a streptavidin (SA)-biotin backbone that was shown to hasten the immunogenicity of the peptides. The presence of antibodies against HCMVpp65 and TAF9 proteins was also examined in human subjects with SLE, those with other autoimmune diseases, and normal controls to confirm the findings in humans.

## Results

### The correlation between anti-HCMVpp65_422-439_ and anti-TAF9 activities in SLE

**Figure 1:**
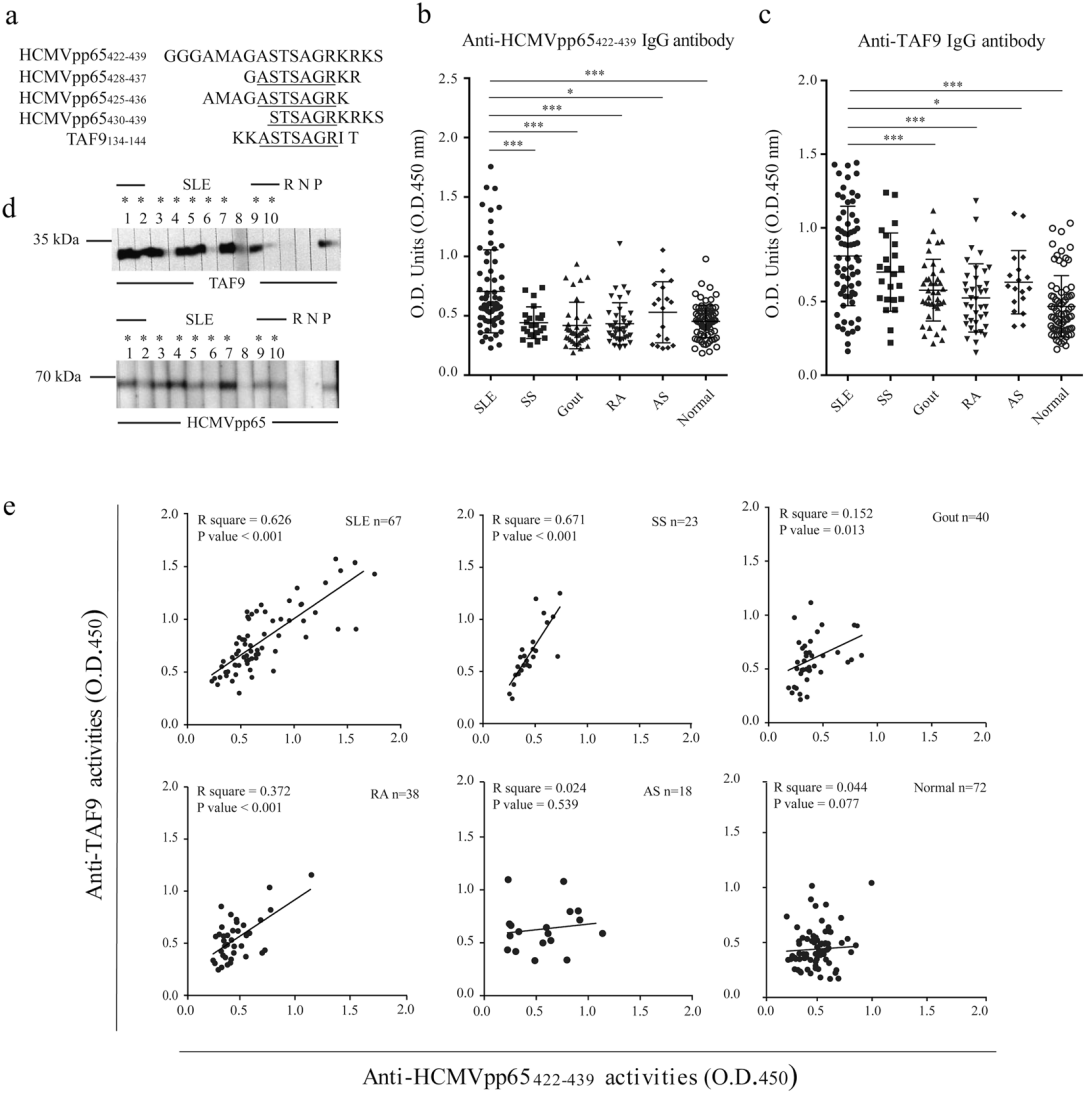
Association between anti-HCMVpp65_422-439_ and anti-TAF9 activities in SLE. (**a**) Alignment of the amino acid sequences for HCMVpp65_422-439_, HCMVpp65_425-436_, HCMVpp65_428-437_, HCMVpp65_430-439_ and TAF9_134-144_. HCMVpp65_425-436_ and HCMVpp65_428-437_ B cell epitopes were recognized by purified anti-HCMVpp65_422-439_ IgG from the sera of human SLE, and HCMVpp65_422-439_ immunized mice, respectively. The TAF9 fragment contains a TAF9_136-142_ epitope like HCMVpp65_428-437_ (**b**,**c**) ELISA analysis for the detection of IgG against HCMVpp65_422-439_ and TAF9 protein using sera from patients with SLE (n = 67), SS (n = 23), RA (n = 38), AS (n = 18), gout (n = 40) and healthy controls (n = 72). (**d**) Western blotting for the examination of IgG antibodies response to HCMVpp65 and TAF9 proteins. There was a positive reaction to HCMVpp65 and TAF9 proteins using dual positive sera in the ELISA test. The positivity was defined as the mean + 3SEM of the sera. Sera with an optical density (O.D.)_450_ > 0.932 of anti-TAF9 activity and O.D._450_ > 0.835 of anti-HCMVpp65_422-439_ activity were considered to be dual positive sera. Asterisks indicate the sera are positive for HCMVpp65 or TAF9. SLE: sera of SLE patients; N and R: serum of normal population and patient with RA, respectively. P: positive control; His-tagged HCMVpp65 or TAF9 protein was recognized by the HRP-conjugated anti-His-tag antibody. Uncropped blots related to Fig. 1 were shown in Supplementary Fig. S4. (**e**) Correlation of anti-HCMVpp65_422-439_ and anti-TAF9 activities in patients with rheumatic diseases and healthy controls. Data are shown as the mean ± SEM of three independent experiments.

The fine mapping of the linear B cell epitope within HCMVpp65_422-439_ has been performed in our previous works^16,17^. HCMVpp65_428-437_ is a dominant epitope to elicit IgG activity in mice at the early immunological stage. The targets of elicited IgG include HCMVpp65_425-434_, HCMVpp65_428-437_, and HCMVpp65_430-439_, which may be the result of epitope spreading^17^. The amino acid composition of HCMVpp65 peptides was showed in Fig. 1a. Therefore, we suppose that HCMVpp65_428-437_ is an immunodominant epitope of HCMVpp65_422-439_ to induce cross-reactive antibodies against viral and host proteins. To investigate whether the linear epitope, HCMVpp65_428-437_, was shared between HCMVpp65 and host proteins, a similarity search was conducted using the human amino acid sequence and the BLASTP program. Five candidate proteins were identified, but the fragment of TATA-box binding protein associated factor 9 (TAF9)_134–144_ exhibited the highest alignment score, lowest E-value, and shared similar identity with HCMVpp65_428-437_ (70%, see Supplementary Table S1). We hypothesized that the sequence homology between viral peptide HCMVpp65_428-437_ and host nuclear proteins might lead to the development of autoantibodies against TAF9 as a result of molecular mimicry.

We enrolled 67 SLE patients and 119 disease controls (primary Sjögren’s syndrome [SS], n = 23; rheumatoid arthritis [RA], n = 38; ankylosing spondylitis [AS], n = 18; gout, n = 40) and 72 healthy individuals. Their anti-HCMVpp65_422-439_ and anti-TAF9 IgG antibodies were examined to verify the association between anti-HCMVpp65422-439 and anti-TAF9 IgG antibody (Table 1). A significantly higher anti-HCMVpp65_422-439_ antibody titer was observed in SLE patients (0.706 ± 0.043) compared with individuals with SS (*p* < 0.001), RA (*p* < 0.001), AS, (*p* = 0.049), gout (*p* < 0.001) and healthy controls (*p* < 0.001; Fig. 1b). This result is consistent with our previous findings^17^. Sera from patients with SLE exhibited a significantly higher titer of anti-TAF9 IgG antibody compared with the sera from patients with RA (*p* < 0.001), AS (*p* = 0.037), gout (*p* < 0.001) and healthy individuals (*p* < 0.001), No statistically significant difference was detected when the sera were compared with those from the SS cohort (*p* = 0.163; Fig. 1c). To confirm the ELISA results, sera (10 SLE, 1 RA, and one healthy control) that were dual positive for HCMVpp65_422-439_ and TAF9 were validated by western blot (Fig. 1d). In total, 9/10 SLE sera reacted to the full-length human TAF9 and HCMVpp65 proteins, but neither the normal nor RA sera exhibited a positive antibody response. The positive association between anti-TAF9 and anti-HCMVpp65_422-439_ antibody activities was significant in SLE, SS, gout, and RA; however, most sera from SS, gout, or RA patients had a poor response to HCMVpp65_422-439_ or TAF9 in the ELISA test (Fig. 1e).

**Table 1:** Characteristics of the enrolled study participants.

Characteristics	SLE	SS	RA	AS	Gout	Normal
Age (years)	19-73	23-78	22-80	20-72	27-77	32-64
Mean (years)	40.7 ± 1.5	50.41 ± 2.7	52.5 ± 2.0	43.5 ± 3.4	53.8 ± 2.1	43.2 ± 2.1
Total no. of specimen	67	23	38	18	40	72
Female (%)	95.5%	91.3%	65.8%	22.2%	0%	100%

RA, rheumatoid arthritis; AS, ankylosing spondylitis; SS, primary Sjögren’s syndrome; SLE, systemic lupus erythematosus.

### HCMVpp65_422-439_ and TAF9_134-144_ immunization induced cross-reactive antibodies to nuclear antigens and dsDNA

**Figure 2:**
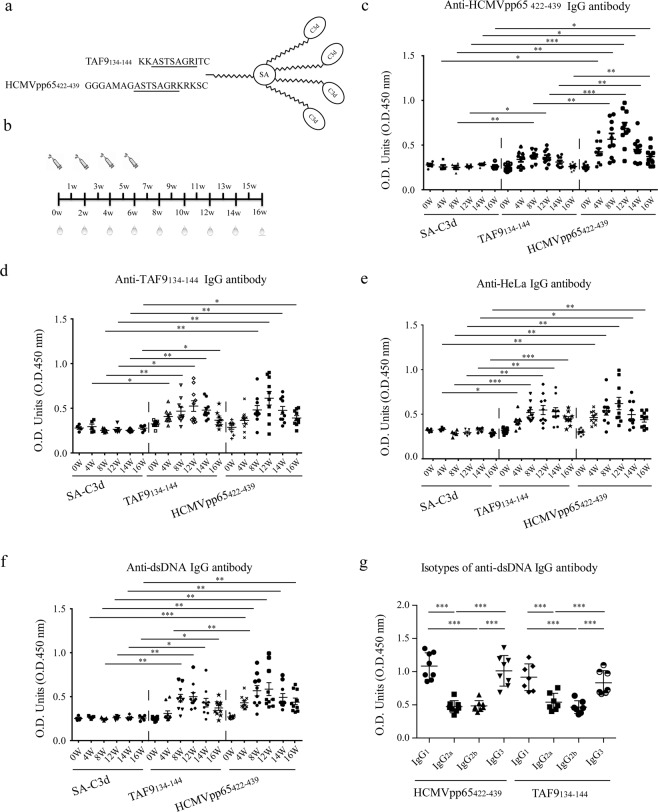
Detection of cross-reactive IgG antibodies against immunized peptides and cellular proteins and dsDNA in the sera of HCMVpp65_422-439_, TAF9_134-144,_ and SA-C3d immunized mice. (**a**) Schematic diagram of the peptide-C3d complex. **(b)** Diagram of the immunization schedule. **(c**–**f)** ELISA analysis for anti-HCMVpp65_422-439_, anti-TAF9_134-144_, anti-HeLa lysate and anti-dsDNA activity from the sera of HCMVpp65_422-439_ (n = 10), TAF9_134-144_ (n = 10) and SA-C3d (n = 5) immunized mice. 250x diluted sera and 1 μg/well HCMVpp65_422-439_ peptide or TAF9 protein were used. (**g**) ELISA analysis for IgG subclasses of anti-dsDNA antibodies from HCMVpp65_422-439_ (n = 8) or TAF9_134-144_ (n = 7) immunized mice at 12 weeks after immunization at 1:80 dilution. Data are shown as the mean ± SEM of three independent experiments.

The role of the humongous sequence in the induction of cross-reactive antibodies against nuclear antigens was examined using a murine model, which immunized BALB/c mice with peptide antigens bound to maleimide-activated streptavidin (SA) complexed with four biotinylated C3d subunits (Fig. 2a,b)^17^. BALB/c mice immunized with HCMVpp65_422-439_-C3d or TAF9_134-144_-C3d (in the following referred to as HCMVpp65_422-439_ or TAF9_134-144_ immunized mice) had an elevated titer of anti-HCMVpp65_422-439_ or anti-TAF9_134-144_ IgG antibodies four weeks after immunization (Fig. 2c,d). Sera from HCMVpp65_422-439_ immunized mice recognized both peptides; however, TAF9_134-144_ immunized sera poorly reacted with HCMVpp65_422-439_, compared with the reactivity of HCMVpp65_422-439_ immunized sera against TAF9_134-144_.

The antinuclear antibody (ANA) and anti-dsDNA antibody are hallmark serological features of SLE. ELISA analysis and indirect immunofluorescence assay (IFA) were performed to determine the ANA profile in HCMVpp65_422-439,_ and TAF9_134-144_ immunized mice. Immunization with HCMVpp65_422-439_ or TAF9_134-144_ elicited an anti-HeLa IgG response, which began at four weeks and peak at 12 weeks after immunization (Fig. 2e). Many different anti-nuclear antibody patterns can be identified in mice at 12 weeks after immunization with HCMVpp65_422-439_ and TAF9_134-144_, including nucleosome/chromatin, speckle/nuclear dots, MSA-II, centrioles, nuclear rim, and cytoplasmic proteins (see Supplementary Fig. S1 and Supplementary Table S2). Immunization with SA-C3d alone induced a low titer nuclear dots pattern, which was detected at 1:20 dilution. Nuclear staining was not observed in SA-C3d mice at 1:100 dilutions or higher. Antibodies against cytoplasmic components were identified in HCMVpp65_422-439_ immunized mice (7/10), and TAF9_134-144_ immunized mice (2/10).

The presence of anti-dsDNA antibodies was tested using ELISA and *C. luciliae* assay. The semi-quantitative ELISA assay demonstrated that both HCMVpp65_422-439_ and TAF9_134-144_ immunized mice exhibited significantly higher titers of IgG against dsDNA compared with SA-C3d immunized mice four weeks after immunization (Fig. 2f). Three serial dilutions (1:20, 1:40 and 1:80) were further performed for the *C. luciliae* assay at 4, 8, 12, 14, 16 weeks after immunization (see supplementary Fig. S2a and supplementary Table S3). Anti-dsDNA activities were not found in SA-C3d immunized mice at dilutions of 1:40 or 1:80, while the HCMVpp65_422-439_ and TAF9_134-144_ immunized group had positive findings at four weeks at 1:40 (7/10, 5/10) and 1:80 (4/10, 2/10) dilutions, respectively. None of the mice with SA-C3d demonstrated any anti-dsDNA activity at 8–16 weeks after immunization. At 12 weeks after immunization, HCMVpp65_422-439_ immunized mice were observed a rise in anti-dsDNA level [1:20 (9/10), 1:40 (9/10) and 1:80 (8/10)]; TAF9_134-144_ immunized mice also resulted in anti-dsDNA serum activity [1:20 (8/10), 1:40 (7/10) and 1:80 (7/10)] after immunization. IgG_1_ and IgG_3_ were the dominant anti-dsDNA antibodies in both groups (Fig. 2g). The number of animals positive for dsDNA at 1:80 serum dilutions is reported in supplementary Fig. S2b. These results demonstrated that HCMVpp65_422-439_ and TAF9_134-144_ immunization induced antibodies reactive with cellular proteins and dsDNA.

### Affinity Purified anti-HCMVpp65_422-439_ and anti-TAF9_134-144_ antibodies from sera of immunized mice and human SLE patients recognized dsDNA

**Figure 3:**
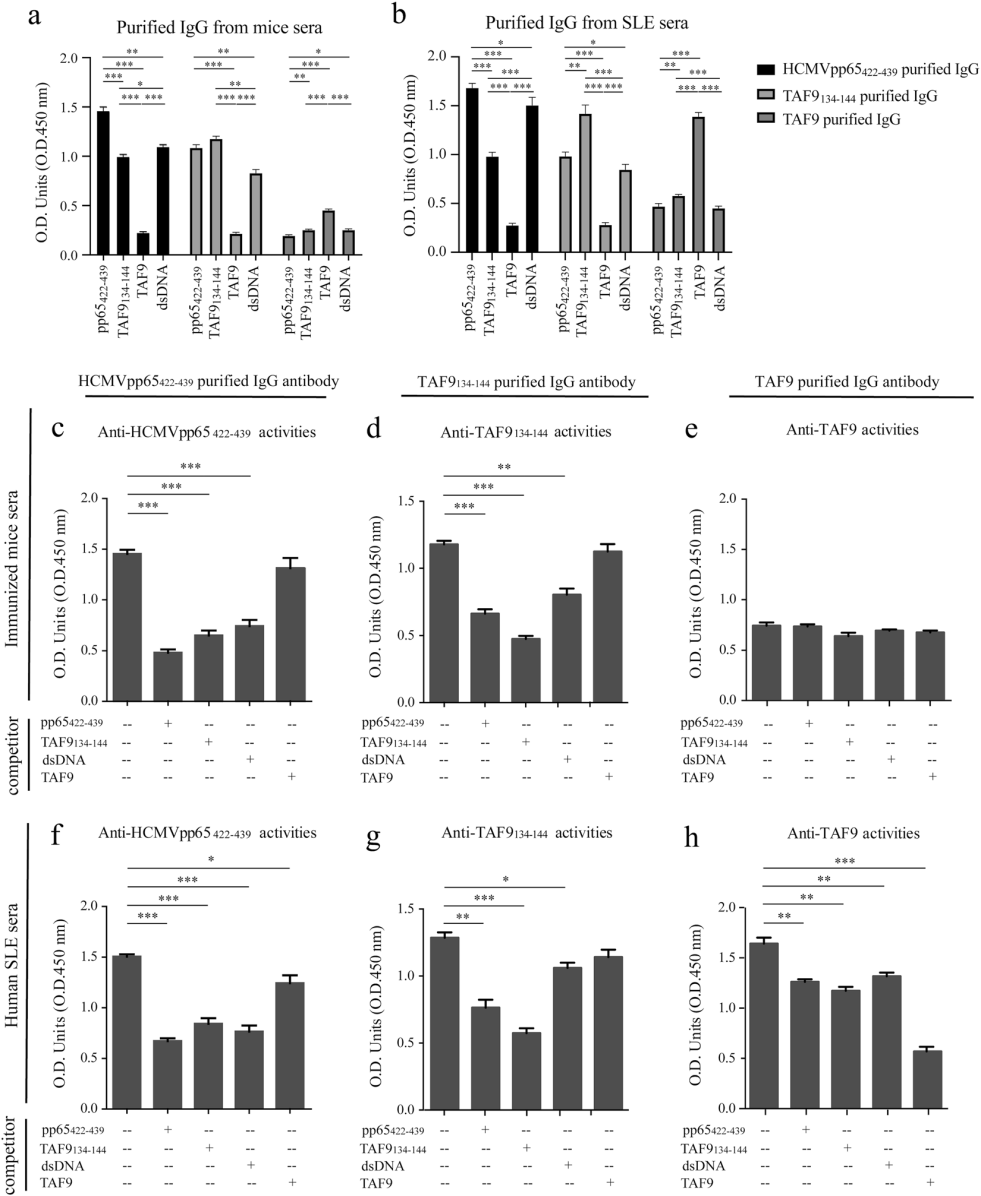
ELISA analysis for HCMVpp65_422-439_, TAF9_134-144,_ and TAF9-specific IgG purified from pooled sera of immunized mice at 10-12 weeks after immunization or patients with SLE. ELISA analysis for anti-HCMVpp65_422-439_, anti-TAF9_134-144_, anti-TAF9, and anti-dsDNA activities using purified IgG from (**a**) immunized mice sera and (**b**) human SLE sera. A total of 1 μg anti-HCMVpp65_422-439_ or anti-TAF9_134-144_ IgG antibody, or 100 μl eluted anti-TAF9 IgG fraction (1 ml/tube) was used. ELISA competitive analysis for anti-HCMVpp65_422-439_, anti-TAF9_134-144,_ and anti-TAF9 activities using purified IgG from the sera of **(c**–**e)** immunized mice and (**f**–**h**) human SLE sera. For the competitive assay, 2 μg/well HCMVpp65_422-439_, TAF9_134-144_, dsDNA or TAF9 protein was used as competitor agents. Data are shown as the mean ± SEM of three independent experiments.

To further examine the association of HCMVpp65_422-439_ and TAF9_134-144_ IgG antibodies, we performed the affinity chromatography to purify anti-HCMVpp65_422-439_, anti-TAF9_134-144_, and anti-TAF9 IgG antibodies from pooled sera of immunized mice at 10–12 weeks post-immunization and human SLE. ELISA analysis for anti-HCMVpp65_422-439_, anti-TAF9_134-144_, anti-TAF9, and anti-dsDNA activity was carried out using IgG eluted fraction. HCMVpp65_422-439_ and TAF9_134-144_ IgG antibodies either purified from human SLE or immunized mice reacted with HCMVpp65_422-439_, TAF9_134-144,_ and dsDNA (Fig. 3a,b). Only a small amount of anti-TAF9 IgG antibodies were purified from mice sera. The mouse anti-TAF9 IgG antibodies had poor reactivity with HCMVpp65_422-439_, TAF9_134-144,_ and dsDNA (Fig. 3a). In contrast, anti-TAF9 IgG antibodies purified from SLE sera reacted weakly with HCMVpp65_422-439_, TAF9_134-144,_ and dsDNA (Fig. 3b).

For competitive ELISA analysis, HCMVpp65_422-439_, TAF9_134-144_, TAF9 or dsDNA as competitor agent was added respectively in each reaction well. In the animal study, the binding capacity of HCMVpp65_422-439_ and TAF9_134-144_ purified mouse IgG was inhibited or partially inhibited by HCMVpp65_422-439_, TAF9_134-144,_ or dsDNA, but not by the TAF9 protein (Fig. 3c,d). The addition of HCMVpp65_422-439_, TAF9_134-144,_ or dsDNA had no apparent suppressive effect on the anti-TAF9 activity (Fig. 3e). Similarly, in the human study, reactions between purified IgG against HCMVpp65_422-439_, TAF9_134-144,_ or TAF9 and their respective targets were suppressed by HCMVpp65_422-439_, TAF9_134-144,_ dsDNA, and TAF9. However, the competitive analysis of TAF9 in anti-TAF9_134-144_ activity was not significant (Fig. 3f,g). Interestingly, the partial suppression mediated by HCMVpp65_422-439_, TAF9_134-144,_ and dsDNA were observed in anti-TAF9 antibody activities (Fig. 3h).

### Immunization with HCMVpp65_422-439_ or TAF9_134-144_ induced early signs of SLE

**Figure 4:**
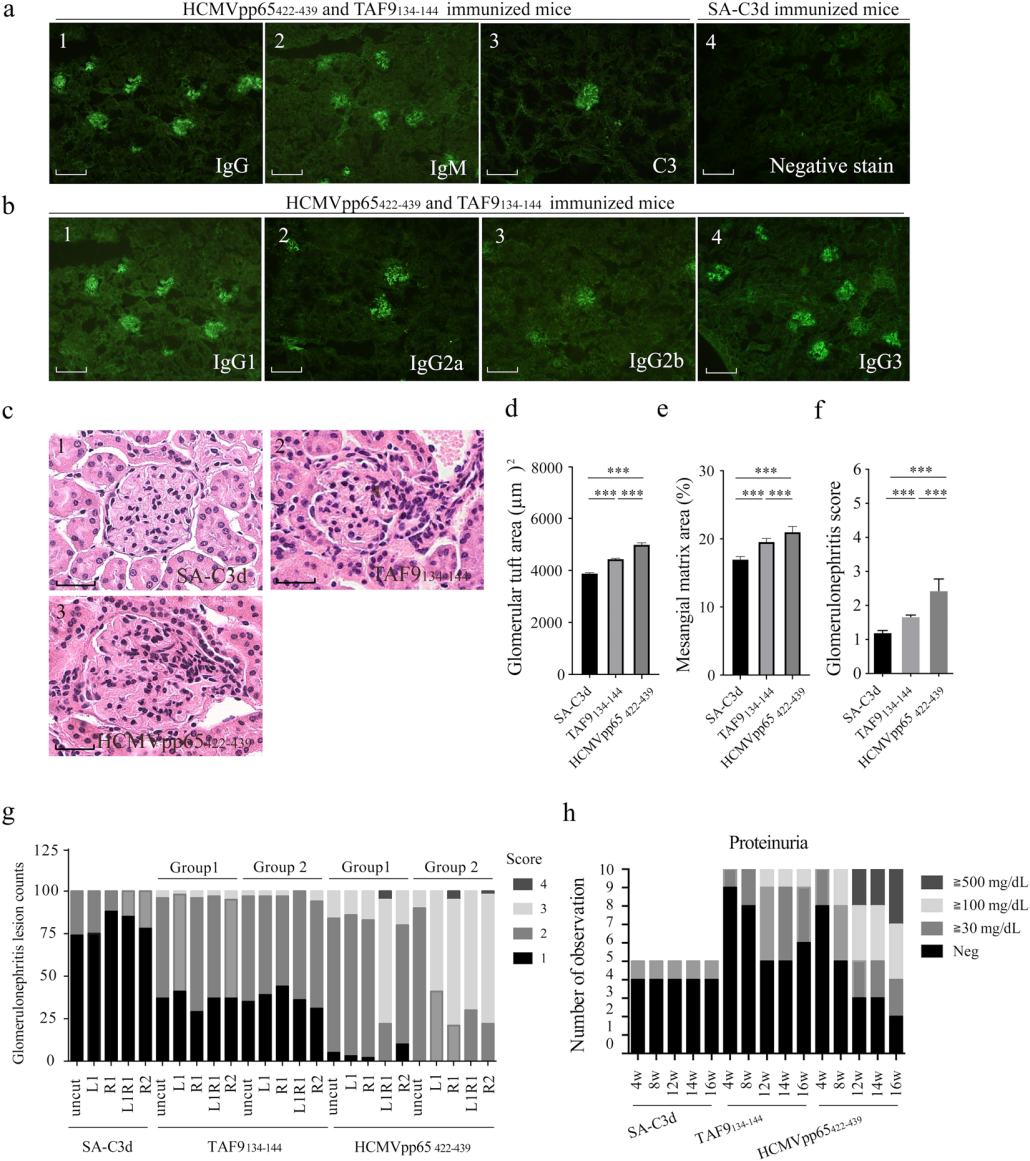
Investigating IgG deposition and the histopathology of glomeruli from HCMVpp65_422-439_, TAF9_134-144_, and SA-C3d immunized mice at 16 weeks after immunization. (**a**) Kidney sections from HCMVpp65_422-439_ and TAF9_134-144_ immunized mice were stained with FITC-conjugated anti-mouse (**a1**) IgG, (**a2**) IgM and (**a3**) C3. Immunoglobulin deposition was not found in glomeruli from (**a4**) SA-C3d immunized mice. Scale bar represents 100 nm **(b)** Kidney sections from HCMVpp65_422-439,_ TAF9_134-144,_ and SA-C3d immunized mice at 16 weeks post-immunization were stained with FITC-conjugated anti-mouse **(b1)** IgG1, (**b2**) IgG2a (**b3**) IgG2b and (**b4**) IgG3. Scale bar represents 100 nm. **(c)** Hematoxylin and eosin staining of the glomerular from (**c1**) SA-C3d (**c2**) TAF9_134-144_ and (**c3**) HCMVpp65_422-439_ immunized mice. Scale bar represents 35 nm. (**d**) Glomerular tuft area. Glomerular hypertrophy is evaluated in three groups of mice, (**e**) Mesangial matrix expansion index. The ratio of expanded mesangial surface area to the total glomerular surface area. (**f**) The glomerulonephritis score of renal lesions from immunized mice. (**g**) Glomerulonephritis lesion counts. One hundred glomeruli per mouse were counted without overlapping. Ear holes produced by an ear punch device are used to identify individual mouse. L: left ear; R: right ear. (**g**) Mice developed proteinuria at 4, 8 12, 14, and 16 weeks after immunization. Data are shown as the mean ± SEM of three independent experiments. The full images related to Fig. 4 were shown in Supplementary Fig. S4.

The immunized mice were sacrificed at 16 weeks after immunization. Their kidneys were taken from immunized mice and examined for immune complex-mediated nephritis using immunofluorescence and hematoxylin & eosin staining. HCMVpp65_422-439_ or TAF9_134-144_ immunized mice had intense IgG (8/10 & 4/10), IgM (7/10 & 2/10) and C3 (4/10 & 3/10) deposition in their glomeruli, respectively (Fig. 4a). IgG_1_ (8/10 & 4/10) and IgG_3_ (8/10 & 4/10) were the most dominant in the glomerular deposits of HCMVpp65_422-439,_ and TAF9_134-144_ immunized mice (Fig. 4a,b and see Supplementary Table S4). No complement or Ig deposition staining was observed in the SA-C3d immunized mice. To evaluate the renal damage, the number of specific abnormalities in 100 glomeruli in a 5-μm-thick H&E-stained paraffin section from each kidney was recorded. The glomerular abnormality was scored in following categories: normal glomeruli (score 1), pure mesangial alterations (score 2), focal segmental glomerulonephritis (score 3), diffuse glomerulonephritis (score 4), Diffuse membranous glomerulonephritis (score 5) and advanced sclerosing glomerulonephritis (score 6), which are based on the 1982 classification published under the World Health Organization^18^. Mesangial alternation and moderate hyper-cellularity were observed in the glomeruli of HCMVpp65_422-439,_ or TAF9_134-144_ immunized mice (Fig. 4c). The area of glomerular tuft and mesangial matrix were estimated by thirty HE-stained glomeruli from mice with the highest glomerulonephritis score in each group (Fig. 4d,e). The mice immunized with HCMVpp65_422-439_ exhibited a higher number of affected glomeruli and increased glomerulonephritis scores for renal lesions compared with TAF9_134-144,_ or SA-C3d immunized mice (Fig. 4f,g). Urine protein analysis was performed at 4, 8, 12, 14, and 16 weeks after immunization (Fig. 4h). At the end of follow-up, proteinuria>100 mg/dL was observed in HCMVpp65_422-439_ immunized mice (6/10), and TAF9_134-144_ immunized mice (1/10).

**Figure 5:**
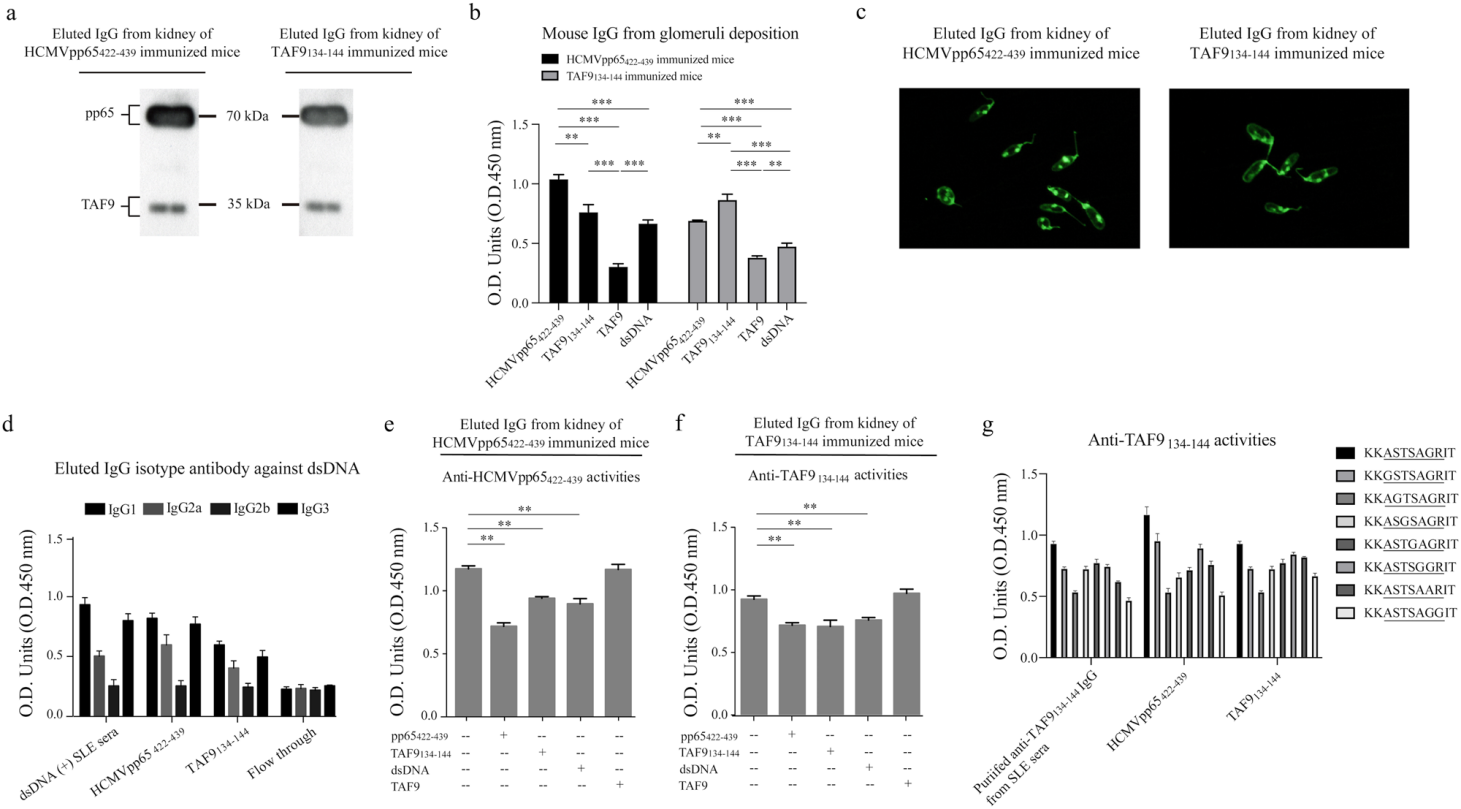
Identification of IgG antibody deposition in the glomeruli of immunized mice. (**a**) Western blot analysis of IgG eluted from the glomeruli of HCMVpp65_422-439_ and TAF9_134-144_ immunized mice against full-length HCMVpp65 or TAF9 protein. (**b**) ELISA analysis for anti-HCMVpp65_422-439_, anti-TAF9_134-144_, anti-TAF9, and anti-dsDNA activities using mouse IgG purified from glomerular deposition. 1 μg of eluted anti-HCMVpp65_422-439_ or TAF9_134-144_ IgG antibody was used for the test. **(c)** Representative images of *Crithidia luciliae* staining with 0.5 μg eluted IgG. Full images/blots were shown in Supplementary Fig. S4 **(d)** Isotypes of eluted IgG against dsDNA. Flow-through was used as the negative control. **(e**–**f)** ELISA competitive analysis for anti-HCMVpp65_422-439_ and anti-TAF9_134-144_ antibody activities using eluted IgG from the glomeruli of immunized mice. For the competitive assay, 2 μg/well of HCMVpp65_422-439_, TAF9_134-144_, dsDNA, or TAF9 protein was used as a competitor. (**g**) Mapping of critical amino acid residues on TAF9_136-142_ peptide. 1 μg of amino acid substituted synthetic peptides, and 1 μg eluted IgG antibodies from glomeruli of HCMVpp65_422-439,_ or TAF9_134-144_ immunized mice, and IgG purified from SLE sera was used for the test. Data are shown as the mean ± SEM of three independent experiments.

To determine the binding activity of IgG deposited in glomeruli, glomerular isolation, and affinity purification of IgG from the glomeruli of HCMVpp65_422-439_ or TAF9_134-144_-immunized mice was performed. The examination of anti-HCMVpp65_422-439_, TAF9_134-144_, TAF9, and dsDNA activity was conducted using eluted IgG fractions. As shown in western blot results, both eluted IgG fraction exhibited anti-HCMVpp65 and anti-TAF9 activities (Fig. 5a). The reactivity of eluted IgG against HCMVpp65_422-439_, TAF9_134-144,_ or dsDNA was detected by the ELISA test and *C. luciliae* assay (Fig. 5b,c). The eluted IgG from TAF9_134-144_ immunized mice showed the weak antibody response to TAF9 protein. Notably, IgG_1_ and IgG_3_ were the major subclasses of anti-dsDNA IgG, which was found in human SLE sera as well (Fig. 5d). For the competitive assay, the ELISA analysis revealed that the partial repression mediated by HCMVpp65_422-439_, TAF9_134-144,_ and dsDNA (but not by TAF9) was observed in anti-HCMVpp65_422-439_ and anti-TAF9 IgG activity (Fig. 5e,f). To map the critical amino acid residues in TAF9_136-142_, seven glycine (G)- or alanine (A)- substituted peptides were used to measure antibody-binding capacity by ELISA test using eluted mouse IgG and purified anti-TAF9_134-144_ IgG from SLE sera. We found that the IgG purified from human or eluted from glomeruli of mice recognized a similar epitope on TAF9_134-144_ peptides (Fig. 5g). As the serine 136 or arginine 142 was replaced by glycine, an apparent reduction of anti-TAF9_134-144_ activity was found in the ELISA test.

## Discussion

The production of various autoantibodies is a hallmark of SLE. Molecular mimicry and epitope spreading are critical mechanisms underlying the development of autoimmunity. The current study used computational analysis to identify sequence homology between HCMVpp65_428-437_ and TAF9_136-142_ and then confirmed its effects on immune regulation using a murine model. For this animal model, BALB/c mice were immunized with either HCMVpp65_422-439_ or TAF9_134-144_, and then induced autoantibodies against HCMVpp65, TAF9, nuclear proteins, and dsDNA was determined. The glomerulonephritis characteristics of SLE were also assessed. Although TAF9_134-144_ immunization induced a weaker humoral response compared with HCMVpp65_422-439_, IgG/C3 deposition and proteinuria were demonstrated in animals immunized by HCMVpp65_422-439_ or TAF9_134-144_. In addition, the competitive analysis revealed a positive association between peptide-induced antibodies and IgG deposition in the kidney glomeruli of mice. In the human study, both anti-HCMVpp65_422-439_ and anti-TAF9 autoantibodies were found in sera from patients with SLE. These results suggest that amino acid similarity between viral and host proteins promotes epitope spreading and accelerates the production of autoantibodies, which may, in turn, induce glomerulonephritis.

Several autoimmune disorders have been reported to link to the CMV infection, especially Sjögren’s syndrome^1,19^. The characteristics of Sjögren’s syndrome can be found in clinical diagnostics and experimental animals as a result of CMV infection that induces circulating autoantibodies against nuclear components and erythrocytes^20,21^. MCMV infection can induce anti-Ro/La antibodies and salivary gland inflammation in C57/BL6^lpr/lpr^ mice^22^. Likewise, HCMV infection elicits the expression of Ro antigen (60 KD/Ro, 52 KD/Ro) on the surface of keratinocyte and anti-phospholipid antibody production^23–25^. Also, HCMV early RNA has been demonstrated to induce antibodies against Ro antigen (SSA)^24^. In the current study, we enrolled patients with Sjögren’s syndrome to determine the correlation between anti-HCMVpp65_422-439_ and anti-TAF9 antibodies. The ELISA analysis showed the low titer of anti-HCMVpp65_422-439_ antibody detected in patients with SS, suggested the epitope of HCMVpp65_422-439_ did not involve in the development of Sjögren’s syndrome.

Many studies of HCMV have researched on the HCMV Towne and AD169 strains and focused on their potential host-virus interaction through efficient replication in human fibroblasts^26,27^. During HCMV infection, HCMVpp65 is transported into the nucleus mediated by two nuclear localization signals (HCMVpp65_418-438_ and HCMVpp65_537-561_) on C-terminus of HCMVpp65^28^. Another study revealed that HCMVpp65 binds to metaphase-arrested chromosomes in fibroblasts during infection, implying that HCMVpp65 does not merely bind to host proteins, but also forms immune-complex to nuclear components^29^. A similar finding was found in human polyomaviruses (HPyV) infection. The SV40 large T-antigen of HPyV forms complexes with nucleosome, subsequently is targeted by host immune responses, and elicits the generation of antibodies against both virus and host^30^. These findings may provide the basis of hypothesis that the HCMVpp65 binds to immune complexes to forms from genetic matrix or nuclear components, which may not merely be targets by anti-viral antibodies, but also raises the opportunity for B cell epitope spreading.

HCMVpp65 is an immunodominant T cell determinant in healthy individuals; however, the anti-HCMVpp65_422-439_ antibody was more prevalent in SLE patients and had a higher specificity compared with other rheumatic diseases and healthy controls^17,31^. The current study detected a significantly higher titer of anti-HCMVpp65_422-439_ antibodies in the SLE cohort than normal cohort or diseased controls. Although some human subjects had autoantibodies against TAF9, it was not possible to detect antibodies reacting to TAF9 by western blot, suggesting that the detected antibody response in the ELISA test may be attributed to the conformation epitope of TAF9 being recognized by the tested sera. Western blot analysis found that 90% of the dual positive sera were positive for HCMVpp65 and TAF9 protein. The cross-reactivity between serum anti-HCMVpp65_422-439_ and anti-TAF9 antibodies in SLE sera implied the possibility that the occurrence of the epitope is spreading between HCMVpp65 and TAF9 proteins during viral infection.

The poor immunogenicity of short peptides is a problem in peptide-induced immune responses, which often requires carrier proteins or molecular adjuvants to bridge innate and adaptive immunity^32^. In the current study, the peptide-murine C3d complex was linked by a streptavidin-biotin backbone through the engagement of the C3d-CR2 and B cell receptor-peptides, which provided a stimulating signal to lower the threshold of T cell-dependent B cell activation^33,34^. The immunization scheme of using a complex of streptavidin-conjugated HCMVpp65_422-439_ or TAF9_134-144_ with biotinylated C3d provoked a vigorous humoral-mediated immune response. Following immunization, HCMVpp65_422-439_ and TAF9_134-144_ immunization elicited antibodies against antigens from HeLa cells and produced ANA stain patterns. The production of multiple autoantibodies is a prominent feature of SLE^35^. However, the humoral immune response was retarded in mice 12 weeks after immunization, suggesting genetic predisposition plays a large role in SLE development.

Anti-dsDNA antibodies are a characteristic autoantibody for SLE, which plays a crucial role in lupus glomerulonephritis. Evidence for antigen selection of anti-dsDNA antibodies has illustrated that the basic or positively charged amino acid residues [arginine (R), asparagine (N) and lysine (K)], prefer to interact with DNA from virus or necrotic cell, and have the potential to induce anti-dsDNA antibodies during clonal expansion and affinity maturation^36^. The ELISA test in the current study demonstrated anti-dsDNA reactivity in HCMVpp65_422-439_ and TAF9_134-144_ purified human antibodies and immunized serum. The HCMVpp65_422-439_ and TAF9_134-144_ immunization not only induced anti-dsDNA antibodies but also initiated early-phase glomerulonephritis in BALB/c mice. In contrast, SA-C3d immunization did not induce ANA or dsDNA reactivity, which suggests that SA-C3d alone is less likely to induce autoimmunity.

Furthermore, TAF9_134-144_ and HCMVpp65_422-439_ specific IgG either from the immunized mice or SLE sera were predominantly made up of IgG_1_ and IgG_3_ isotypes. In human studies, IgG_3_ has been previously found to underlie autoimmune organ damage^37,38^. Anti-glomerular basement membrane (GBM) IgG_3_ deposits along the GBM and tubular basement membrane plays a part in the renal injury of anti-GBM diseases^39^. The formation and deposition of immune complex is a crucial factor exacerbating human lupus nephritis^36,40^. The predominance of human IgG_1_ and IgG_2_ isotypes of anti-nucleohistone and anti-dsDNA antibodies was observed in the plasma of patients with SLE^41^. In the current study, mice immunized with HCMVpp65_422-439_ or TAF9_134-144_ elicited serum titers of anti-dsDNA antibodies, which were positively correlated with the severity of IgG deposition and the severity of proteinuria (see supplementary Fig. S3 and Supplementary Table S5). In addition, IgG eluted from the glomeruli of immunized mice predominately exhibited IgG_1_/IgG_3_ antibodies against the immunizing peptide and dsDNA, suggesting that the pathogenic potential of anti-dsDNA antibodies may be involved in SLE.

The pathogenic role of anti-dsDNA antibodies in SLE has been extensively investigated^42–44^. However, whether anti-dsDNA antibodies cause kidney damage remains controversial. Early studies indicated that nephritogenic mouse anti-dsDNA antibodies could interact with cell surface proteins on glomerular or vascular cells and lead to mesangial expansion and proteinuria^45^. Recently, the deposition of positively charged nucleosomes and proteins on the GBM, have been described as targets for autoantibodies^40,41,46^. The ELISA competitive analyses in the present study revealed that purified IgG from the kidneys of immunized mice recognized immunized peptides and dsDNA, which suggests that HCMVpp65_422-439_ or TAF9_134-144_ induced antibodies could contribute to IgG deposition on glomeruli.

In the ELISA competitive analysis, only a small amount of anti-TAF9 antibodies were purified from mice sera compared to antibodies purified from SLE sera (Fig. 3a,b). The purified anti-TAF9 antibodies either from mouse or human sera had low binding capacity for TAF9_134-144_. We found that purified anti-TAF9_134-144_ or anti-TAF9 antibodies from mouse or human sera were unable to react with (or be inhibited by) TAF9 or TAF_134-144_. To our best knowledge, the peptide/antigen coupled to CNBr-activated beads is one of the possibilities. For antibody purification, TAF9_134-144_ (linear epitope) or TAF9 protein (conformational epitopes on the surface or structure of TAF9) is conjugated to CNBr activated beads in affinity column to selectively capture epitope-specific antibodies from sera. In the analysis of eluted IgG from glomeruli, we found that the eluted IgG from glomeruli of both immunized mice could react with full-length of HCMVpp65 and TAF9 by western blot (Fig. 5a) but showed poor reaction with TAF9 in ELISA (Fig. 5c). We suggested that purified IgG antibodies against TAF9 and TAF9_134-144_ have different epitope specificity on TAF9 protein.

On the other hand, seven amino acid-substituted synthetic peptides were used to examine the antibody activity with eluted IgG antibody from glomeruli of TAF9_134-144_ immunized mice. The ELISA analysis revealed that the eluted IgG exhibited reduced antibody binding capacity for seven peptides. The polar (S137G) or positively charged amino acid (R142G) is more critical than nonpolar amino acid for antibody binding to peptides (Fig. 5g). However, this result cannot fully explain the mechanism of molecular mimicry in the current study, and more detailed experiments are needed for further validation.

In the current study, the immunization scheme was performed in a normal strain of mice, which were immunized by a C3d based adjuvant to enhance the antigenicity of the antigens; however, the humoral response was not sustained. This indicated that genetic factors play a pivotal role in the development or exacerbation of autoimmune diseases. The limitation of this study includes that our experimental design and data were unable to verify the existence of conformational epitopes due to the strategy of searching previously identified sequences, the method of IgG purification and the examination of IgG antibody responses.

## Conclusion

The current study reports a positive association between anti-HCMVpp65_422-439_ and anti-TAF9 antibody reactivity in the sera of SLE patients. Immunization with the viral peptide-C3d complex provoked a strong humoral response in non-autoimmune-prone strains. The immunization of mice with HCMVpp65_422-439_ or TAF9_134-144_ induced cross-reactive antibodies and early signs of glomerulonephritis, which were characteristic of SLE. These results indicate that sequence homology between exogenous antigen and nuclear component is pivotal in the induction of SLE.

## Methods

### Study populations

All patients were recruited from rheumatology clinics in the Linkou branch of Chang Gung Memorial Hospital. The definition of SLE was based on the 1982 and 1997 American College of Rheumatology diagnostic criteria for SLE^47,48^, and a rheumatologist confirmed all diagnoses. The study was approved by the Institutional Review Board of Chang Gung Memorial Hospital, and all patients provided their written informed consent before their inclusion as required by the Declaration of Helsinki (approval numbers #201600798B0 and 201600795B0).

### Bioinformatics analysis

To determine the sequence homology of HCMVpp65_428-437_ peptides with nuclear proteins in humans, HCMVpp65_428-437-GASTSAGRKR_ was input as a query sequence using the BLASTP program in the alpha release 2.8.0+^49,50^. This procedure searched for high scoring sequence alignments between the query sequence and existing sequences in the reference protein database of Homo sapiens. Alignment score expect value and identity are the standard guidelines for evaluating the degree of homogeneity between sequences.

### Mice

Normal 3 to 5-week-old female BALB/c mice were purchased from the National Laboratory Animal Center (NLAC), Taiwan. The mice were housed in a pathogen-free facility with an independent cage ventilation system; the laboratory animal experiment center was approved by the Institutional Review Board of the Chang Gung Medical Foundation (approval number #2017121309).

### Protein expression and purification

The preparation of pET30a-HCMVpp65-C3d and pET30a-murine C3d constructs were as described previously^17^. Plasmid encoding human TAF9 protein was purchased from Origene Technology (TAF9: RC201550, RefSeq: NM_003187.5), and the full sequences were amplified using TAF9 paired primers: (forward, 5′-CGCGGATCCATGGAGTCGGGCAAGATGGCGCCGCT-3′; and reverse, 5′-CGCCTCGAGCAGATTATCATAGTCATCATC-3′). The PCR fragment was purified and ligated into TA cloning vectors (Yeastern Biotech) to generate TA-TAF9. TAF9 gene fragment was then digested by BamHI/XhoI and ligated into pET30a. All the obtained pET30a constructs were confirmed via restriction enzyme digestion and DNA sequencing. Recombinant proteins were over-expressed in *Escherichia coli*, induced with one mM isopropyl β-D-thiogalactoside (Sigma-Aldrich; Merck KGaA, Darmstadt, Germany) and purified by a nickel affinity column.

### Synthetic peptides and antigen preparation

The preparation of synthetic peptides and antigens were followed according to previously described methods^17^. The purity of all synthetic peptides was >99%, as per the manufacturers’ guarantee (GenScript Biotech Corp, New Jersey, USA). The peptides were prepared and stored according to the manufacturer’s recommendations (20 μg/μl). A total of 6 histidines and one cysteine were added to the C-terminus of the peptides as a target, or for crosslinking with the C3d protein. C3d biotinylation (Thermo Fisher Scientific, Inc., Waltham, MA, USA) and maleimide activated streptavidin (Thermo Fisher Scientific, Inc.) conjugation were performed according to the manufacturer’s protocol. All complexes of peptide-C3d were generated and prepared for immunization within four hours before the injection to ensure the stability of the compounds.

### Immunization

Female BALB/c mice (n = 25) were randomly separated into the following three groups: HCMVpp65_422-439_ (n = 10), TAF9_134-144_ (n = 10) and SA-C3d (n = 5). On day 1, the mice received an intraperitoneal injection with 100 μg HCMVpp65_422-439_-C3d, TAF9_134-144_-C3d or SA-C3d (emulsified with complete Freund’s adjuvant [Sigma-Aldrich]), respectively. Boosting was performed with complex antigens in incomplete Freund’s adjuvant (Sigma-Aldrich) on day 14, 28, and 42.

### Sera and urine collection

The mice were bled from the retro-orbital vein sinus one day before each assay and at two-week intervals. Mouse plasma was collected from the blood by centrifugation at 4 °C for 10 minutes at 13,000 rpm. Unused plasma was stored at −80 °C, and the PBS-diluted sera were stored at 4 °C. Urine was collected to analyze proteinuria at the same time point. The course and onset of proteinuria were monitored at 4, 8, 12, 14, and 16 weeks after immunization by using proteinuria strip (Medi-Test Combi 10 VET strip, MACHEREY-NAGEL, Germany).

### ELISA analysis and immunoblotting

Immunoblotting and ELISA were performed as previously described method^17^. For the competitive assays, 2 μg/well HCMVpp65_422-439_, TAF9_134-144_, dsDNA or TAF9 protein as competitor was mixed with 1 μg purified anti-HCMVpp65_422-439_, anti-TAF9_134-144_ IgG, anti-TAF9 antibody or 100 μl eluted anti-TAF9 IgG fraction (1 ml/tube) with sterile PBS up to the final volume of 250 μl and added into peptide coating well for incubation at 37 °C for 2 hours. Following incubation, the microtiter plate was washed four times with TBST (TBS with 0.05% Tween-20) and any bound antibody was detected by horseradish peroxidase (HRP) conjugated anti-human/mouse IgG or anti-mouse IgG subclasses at 1:5,000 dilution (Jackson ImmunoResearch; Catalog code 109-035-088, 115-035-166, 115-035-205, 115-035-206, 115-035-207, 115-035-209) at 37 °C for 2 hours. After washing, TMB (Sigma-Aldrich) was used as the substrate, and HRP activity was measured at 450 nm by a microplate ELISA reader (EZ read 400).

### Anti-nuclear antibody, and *Crithidia luciliae* immunofluorescence staining

The mice sera were tested for ANAs at 1:100 dilution in PBS using a standard ANA test (Diasorin).

DAPI (4′,6-diamidino-2-phenylindole) is used for nuclear visualization. Anti-dsDNA antibody reactivity was examined via an immunofluorescence stain using the *C. luciliae* assay (Diasorin) at 1:20, 1:40, and 1:80 dilutions in PBS according to the manufacturer’s instructions. Mouse antibodies were detected by 100x diluted FITC conjugated anti-mouse IgG/M and IgG subclasses (Jackson ImmunoResearch; Catalogue codes 115-095-166, 115-095-075, 115-095-205, 115-095-206, 115-095-207, 115-095-209). At the end of staining, the slides were mounted using the mounting medium (Diasorin) for further investigation by fluorescence microscopy (Olympus IX73/DP72, cellSens standard software).

### Isolation of the glomeruli

The isolation of glomeruli was carried out using the modified protocol, according to Minoru Takemoto^51^. Briefly, mice at 16 weeks post-immunization were anesthetized by an anesthetic machine vaporizer with isoflurane (3%) and perfused with 5×10^7^ Dynabeads (4.5μm) in 40 ml of PBS through the heart. After mice sacrifice, their kidneys were immediately removed, cut into one-mm^3^ pieces, and digested with collagenase (1.5 mg/ml collagenase A, 100 U/ml deoxyribonuclease I in Hanks’ Balanced Salt Solution [HBSS]) at 37 °C for 30 minutes with gentle agitation. The collagenase digested tissue was gently pressed through a 70 μm cell strainer twice, and the cell strainer was then washed with 10 ml ice-cold HBSS. The filtered cells were then passed through a new cell strainer, and the cell strainer was washed with 10 ml HBSS. The cell suspension was centrifuged at 1,500 rpm at 4 °C for 10 minutes. The supernatant was discarded, and the cell pellet was re-suspended in 1.5 ml HBSS. Finally, glomeruli containing Dynabeads were harvested by a magnetic particle concentrator (Thermo Fisher Scientific, Inc.) for 20 minutes, and then washed at least three times with 5 ml ice-cold HBSS. Kidney tissues were collected and kept at 4 °C for antibody purification.

### Antibody purification

The protocol for antibody purification was performed as previously described with small modifications^17^. Moderated cyanogen bromide (CnBr) powder (Sigma-Aldrich) was activated according to the manufacturer’s protocol. Briefly, 5 mg of 4 tandem repeats of the HCMVpp65_422-439_ (GGGSGGGAMAGASTSAGRKRKS) or TAF9_134-144_ (GGGSKASTSAGRIT) peptides were dissolved in a coupling buffer with activated CnBr gel and gently rotated at 4 °C overnight. The free active groups on CnBr were blocked by 0.1 M Tris-HCl (pH 8.0) at room temperature (RT) for 2 hours. The CnBr gel was washed with two cycles of alternating pH buffer (each cycle consists of a wash with pH 4.0 buffer containing 0.1 M acetic acid, 0.1 M sodium acetate, and 0.5 M NaCl followed by a wash with pH 8.0 buffer with 0.1 M Tris-HCl and 0.5 M NaCl) and once with 10 ml PBS. Then 300 μl of pooled HCMVpp65_422-439_ or TAF9_134-144_ mouse sera in 10 ml ice-cold PBS was then added to the HCMVpp65_422-439_ or TAF9_134-144_-CnBr gel, respectively and rolled at 4 °C overnight. Next, IgG was purified from the mouse glomeruli. Briefly, the glomeruli were homogenized by sonication with 6 M guanidine hydrochloride and 10-4 iodoacetamide. Dynabeads were removed from homogenized glomeruli by a magnetic particle concentrator, the flushed supernatant of homogenized glomeruli was incubated with protein G Sepharose beads and rolled at 4 °C overnight. The protein G beads were then washed three times with ice-cold HBSS and 0.1% NP40. The unbound portion of the sera or protein flow-through was collected and concentrated as the negative control. Bound antibodies were eluted by 1 ml, 0.1 M glycine (pH 2.0). The eluted samples were immediately neutralized with 50 μl neutralizing buffer (1 M Tris-HCl, 2 M NaCl, pH 8.8).

### Kidney immunofluorescence stain

Kidney immunofluorescence stain was performed as previously described^17^. Briefly, when the kidneys were removed from the mice, they were immediately placed in optimal cutting temperature gel, frozen with liquid nitrogen, and stored at −80 °C before their use. The 3-μm-thick frozen sections were stained with FITC conjugated anti-mouse IgM/G and IgG antibodies (Jackson ImmunoResearch Laboratories) at a 1:100 dilution in PBS at RT for 30 minutes in the dark, humidified chamber. After washing, the tissue slides were prepared for further investigation using coverslips with mounting medium (Diasorin). After washing, the tissue slides were prepared for further investigation using coverslips with mounting medium (Diasorin).

### Hematoxylin and Eosin (H&E) staining

Hematoxylin and eosin staining were carried out according to the Cold Spring Harbor protocols with small modification^52^. The frozen sections of tissue and organs were immersed in 100% ethanol for 30 seconds after they were sectioned and then rinsed ten times in double-distilled H_2_O. Slides in the rack were then put into a container filled with hematoxylin for 5 minutes, and the rack was dipped five times into a jar containing 0.1% HCl. After dipping into tap water for 5 seconds, the rack was dipped into a jar containing 0.1% NH_4_OH 5 times and then into tap water five times. The cytoplasm was stained with eosin and dehydrated as follows: eosin for 3 minutes, 100% ethanol with 0.1% acetic acid for five dips, 100% ethanol I for five dips, 100% ethanol II for five dips, acetone I for five dips, acetone II for five dips, xylene I for five dips and xylene II for five dips. After removing any excess water, the slides were mounted and a cover glass was put on the slide for further investigation by microscopy (Olympus IX73/DP72, cellSens standard software). The glomerular surface area and mesangial matrix area were estimated with thirty glomeruli of mouse with highest glomerulonephritis score in each group using NDP view 2 (Hamamatsu, Hamamatsu City, Japan).

### Statistical analysis

The Student paired/unpaired t-test, and Fisher’s two-tailed exact test with graphs depicting the mean ± three standard error of the mean (SEM) was used for comparisons between 2 groups. Multiple comparison corrections and figures were performed using GraphPad Prism software 6.0. Pearson’s correlation coefficient test was used for these comparisons with graphs depicting the R squared and *p*-value. All tests of statistical hypothesis were done on the 2-sided 5% level of significance. Depending on the context, different levels of significance were reported (*P ≤ 0.05; **P ≤ 0.01; ***P ≤ 0.001). All analyses were performed using SAS version 9.4 (SAS Institute).

### Ethics approval and consent to participate

This study was approved by the Institutional Review Board of the Linkou Chang Gung Memorial Hospital (reference numbers 201600798B0 and 201600795B0). Written informed consent was obtained from all participants prior to sample collection. Animal experiments were approved by the Laboratory Animal Committee of the Linkou Chang Gung Memorial Hospital. All experiments were performed with full compliance with all relevant guidelines and regulations.

### Consent for publication

The participants gave their written consent to the use of their clinical samples for data publication.

## Supplementary information

Supplementary Information.
