# Supplementary material for: Human cytomegalovirus pp65 peptide-induced autoantibodies cross-reacts with TAF9 protein and induces lupus-like autoimmunity in BALB/c mice
Source: Sci Rep. 2020 Jun 15;10:9662. doi: 10.1038/s41598-020-66804-1 (PMC7295797; doi:10.1038/s41598-020-66804-1)
Supplement: Supplementary file 1 — Supplementary Information. [file 41598_2020_66804_MOESM1_ESM.docx]

Supplementary information

**Human cytomegalovirus pp65 peptide induced autoantibodies cross-reacts with TAF9 protein** **a­­nd induces lupus like autoimmunity in BALB/c mice**

**Ao-Ho Hsieh^1*^, Chang-Fu Kuo^1,2,3*^, I-Jun Chou^4,5^, Wen-Yi Tseng^6,7^, Yen-Fu Chen^1^, Kuang-Hui Yu^1^ & Shue-Fen Luo^1^**

1. Division of Rheumatology, Allergy and Immunology, Chang Gung Memorial Hospital, Taoyuan, Taiwan
2. Center for Artificial Intelligence in Medicine, Chang Gung Memorial Hospital, Taoyuan, Taiwan
3. School of Medicine, Chang Gung University, Taoyuan, Taiwan
4. Division of Clinical Neurology, School of Medicine, University of Nottingham, Nottingham, UK
5. Division of Paediatric Neurology, Chang Gung Memorial Hospital, Taoyuan, Taiwan
6. Division of Rheumatology, Allergy and Immunology, Chang Gung Memorial Hospital, Keelung, Taiwan
7. Kennedy Institute, University of Oxford, Oxford, UK

* Corresponding author

^＋^ Equal contribution

Correspondence to: Dr Chang-Fu Kuo

Division of Rheumatology, Allergy and Immunology, Chang Gung Memorial Hospital, Taoyuan, Taiwan

Email: zandis@gmail.com; Telephone: +886 (03) 328-1200 #2140

Supplementary Figure S1~S3


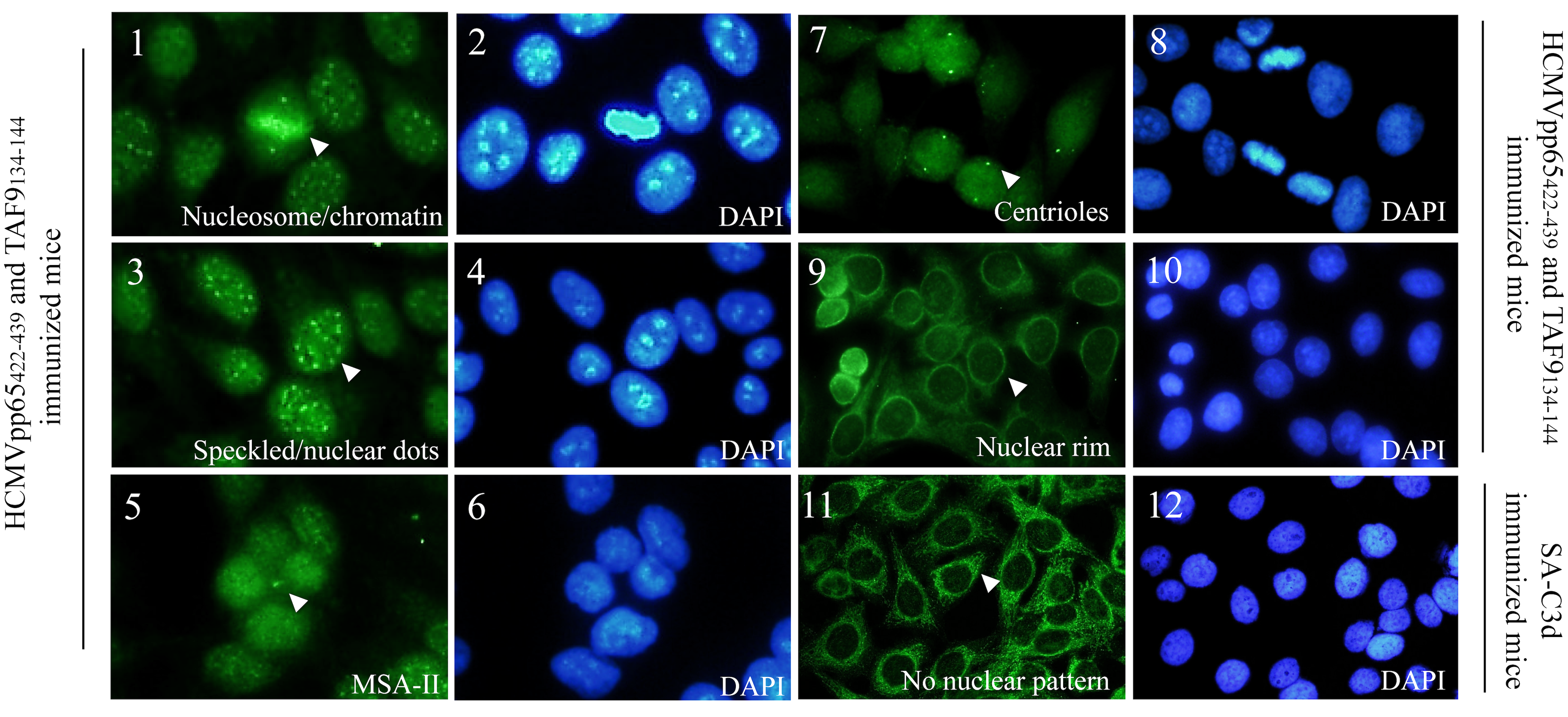


**Supplementary Figure S1.** Detection of anti-nuclear activity in HCMVpp65_422-439_, TAF9_134-144_ or SA-C3d immunized sera. HeLa substrate slides were used for the detection of anti-nuclear antibodies. Sera from 12 weeks post-immunization were diluted 100x for the anti-nuclear antibody (ANA) stains. Patterns of **(1, 2)** nucleosome/chromatin, **(3, 4)** speckled/nuclear dots, **(5, 6)** mitotic spindle type II (MSA-II), **(7, 8)** centrioles and **(9, 10)** nuclear rim were revealed with sera from both HCMVpp65_422-439_ and TAF9_134-144_ immunized mice. Nuclear activity was not found in SA-C3d immunized mice **(11, 12)**. The white arrowheads indicate the pattern of nuclear responses. DAPI is used for nuclear counterstain. W, weeks of post-immunization. The full immunofluorescence images were shown in supplementary Fig.5.


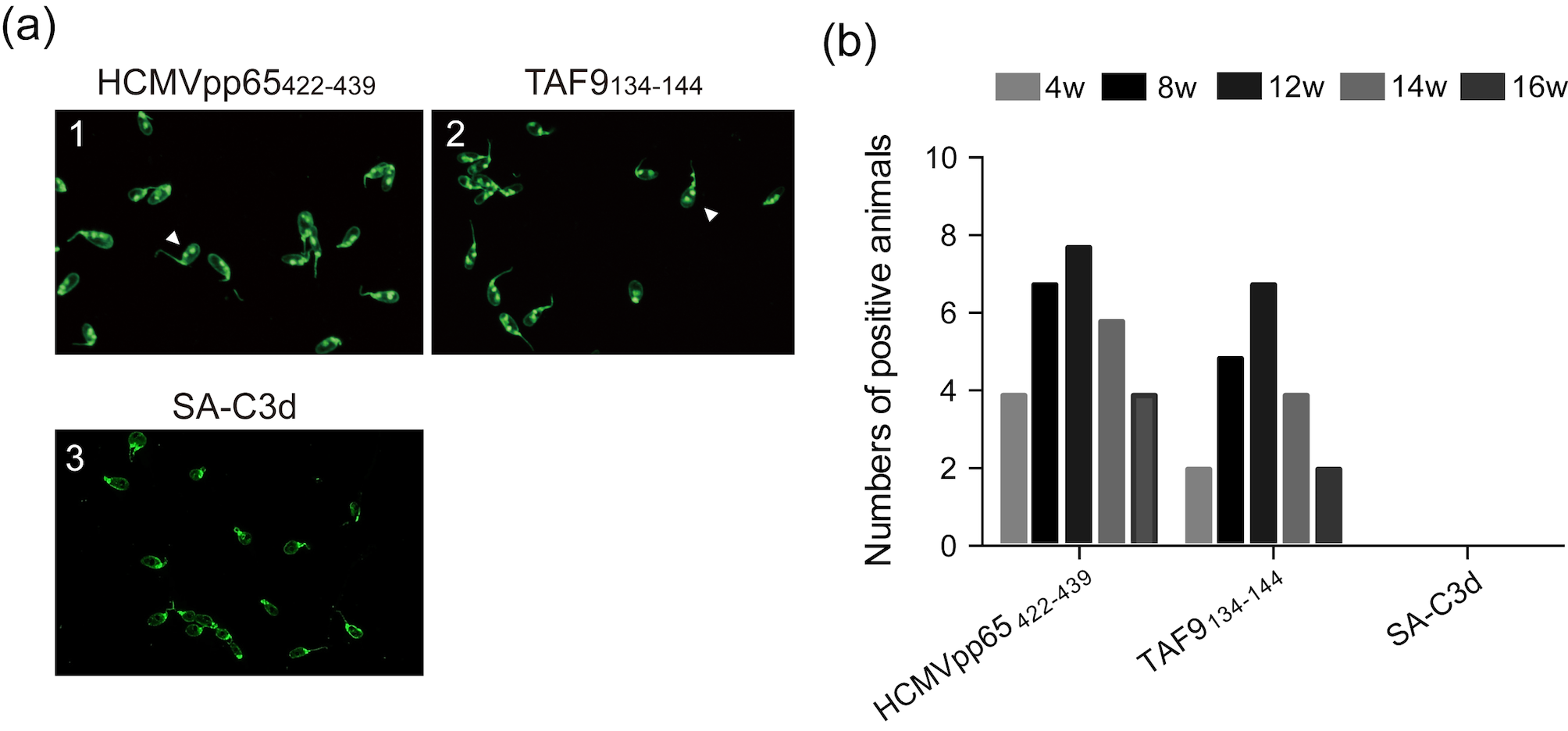


**Supplementary Figure S2.** Detection of anti-dsDNA antibody activity in the sera of HCMVpp65_422-439_, TAF9_134-144_ and SA-C3d immunized mice. (**a**) Representative examples of *Crithidia luciliae*staining of the sera from (**a1**) HCMVpp65_422-439_, (**a2**) TAF9_134-144_, and (**a3**) SA-C3d immunized mice at 12 weeks after immunization at 1:80 dilutions. The white arrowheads indicate dsDNA positive stains. (**b**) The summarized results of *Crithidia lucilia*positive animals at 4, 8, 12, 14 and 16 weeks after immunization at 1:80 dilutions. The full immunofluorescence images were shown in supplementary Fig.5.


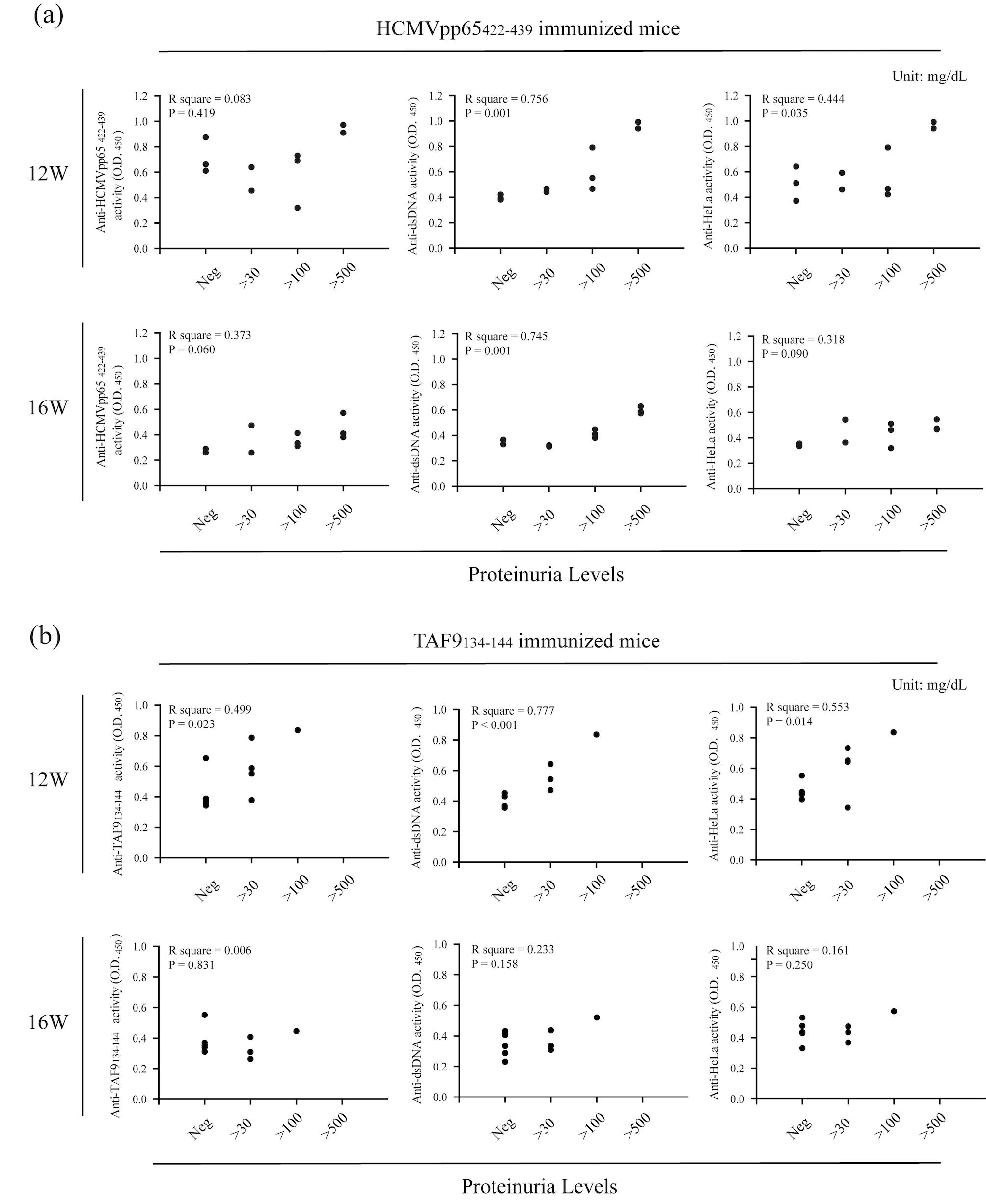


**Supplementary Figure S3**. Correlation of anti-HCMVpp65_422-439_, anti-TAF9_134-144_, anti-HeLa or anti-dsDNA activity and proteinuria levels in immunized mice. (**a**) HCMVpp65_422-439_ immunized mice (**b**) TAF9_134-144_ immunized mice.


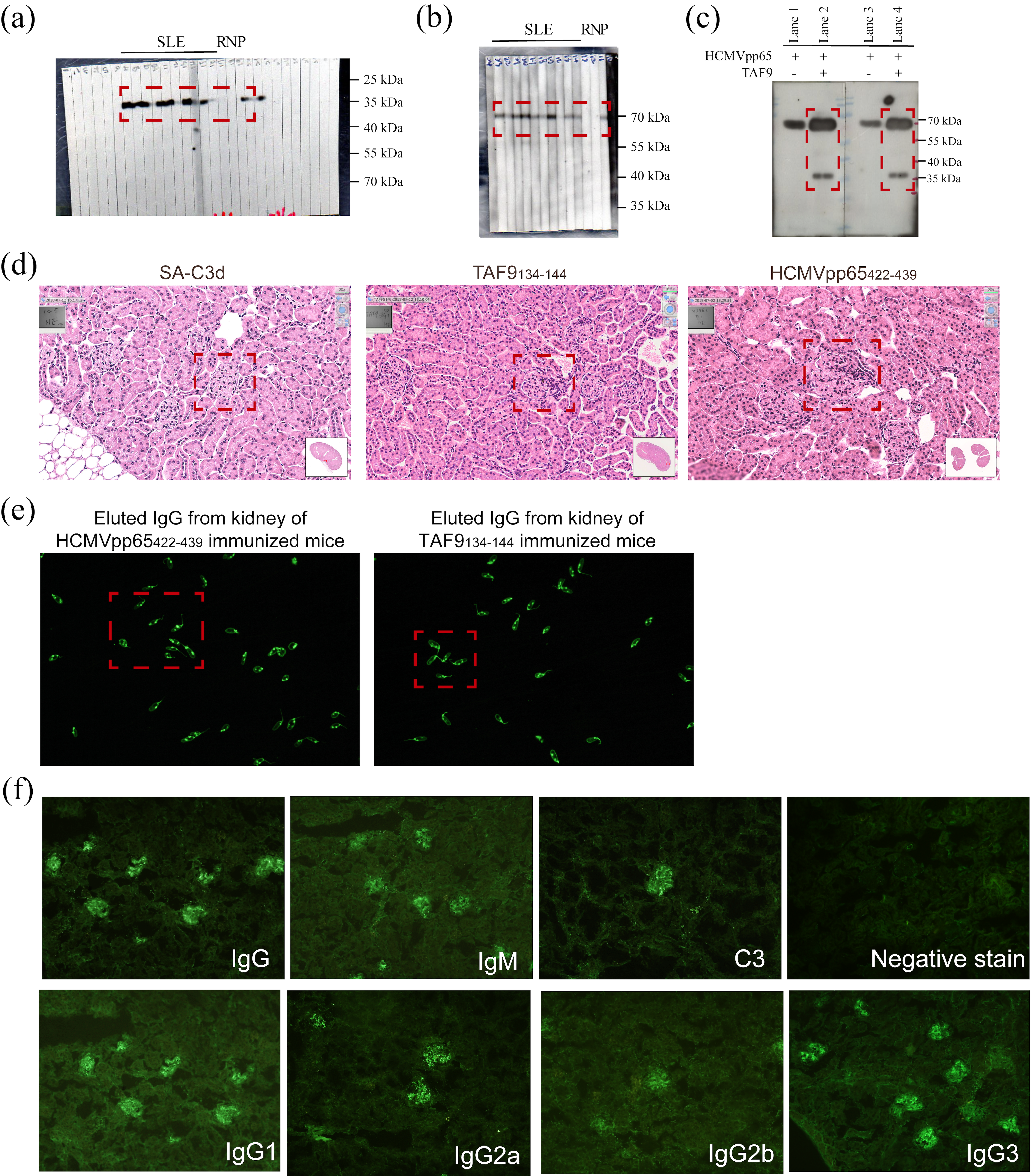


**Supplementary Figure S4**. Full images of western blots, immunohistochemistry staining and immunofluorescence staining. (**a**) TAF9 protein (299 amino acid residues, 32 kDa) and (**b**) HCMVpp65 (561amino acid residues, 65 kDa) blots were used for detection of anti-TAF9 and anti-HCMVpp65 IgG reactivity using sera from SLE, RA (R) and normal healthy (N). 250x diluted sera were used for tests. HRP conjugated mouse anti-His-tag antibody as positive control (P) was used for detection His-tagged pp65 or TAF9 proteins. (**c**) Detection of anti-HCMVpp65 and anti-TAF9 IgG reactivity using IgG antibody eluted from glomeruli of HCMVpp65_422-439_ and TAF_134-144_ immunized mice. (d) Representative of full images of renal tissue sections stained with H&E from SA-C3d, TAF9_134-144_, and HCMVpp65_422-439_ immunized mice. (e) Representative of full images of *crithidia luciliae* stained with IgG eluted from glomeruli of HCMVpp65_422-439_ or TAF9_134-144_ immunized mice. (f) Representative of full images of renal tissue section stained with immunofluorescence to demonstrate Ig/C3 deposits. The square regions in red dashed line are cropped images from full images (magnification 100x field of view, Olympus IX73/DP72, cellSens standard software).


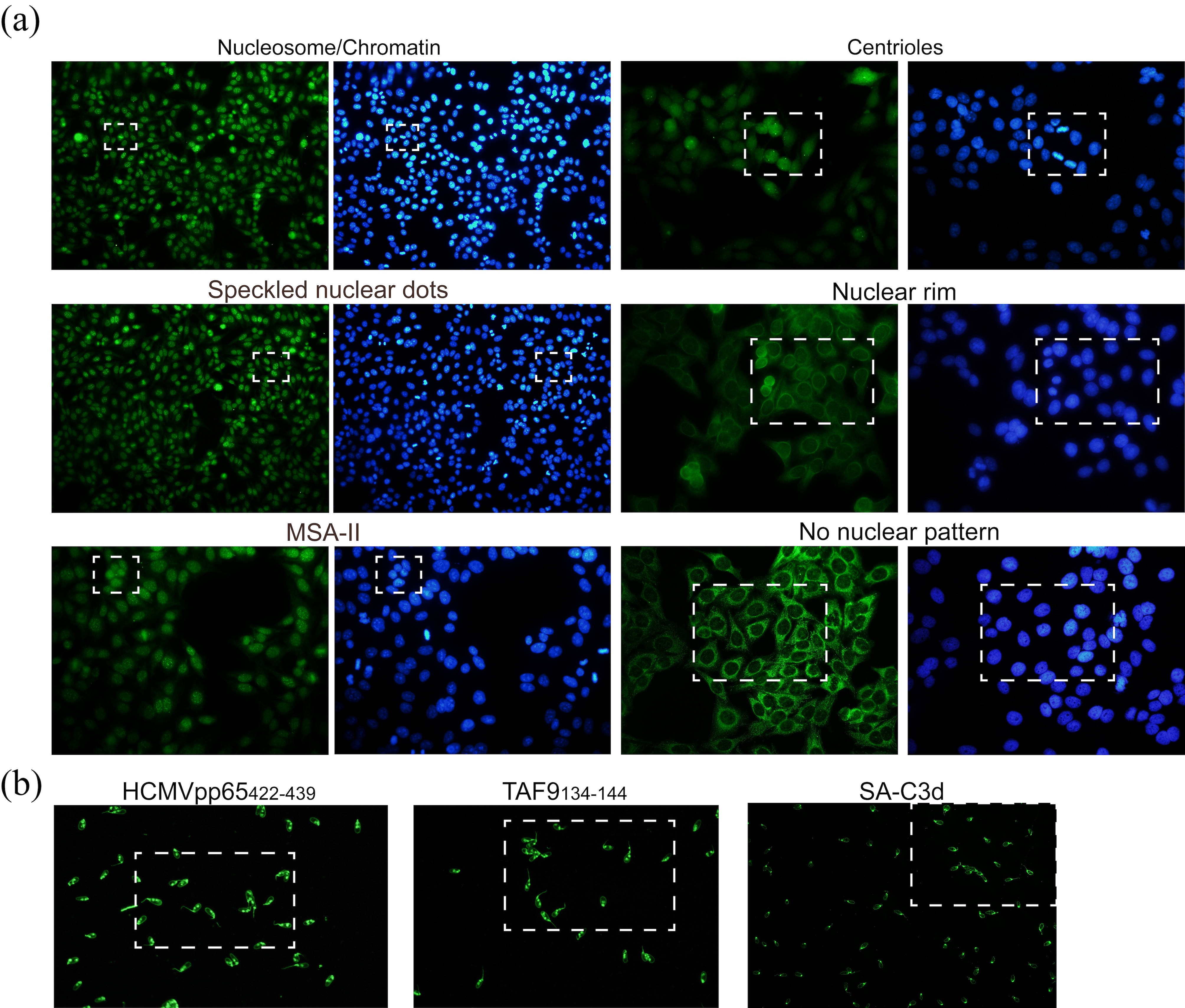


**Supplementary Figure S5**. **Full images of immunofluorescence staining**. **(a)** Representative of full images of antinuclear antibody (ANA) patterns and **(b)** *crithidia luciliae* immunofluorescence tests using sera of HCMVpp65_422-439_, TAF9_134-144_ or SA-C3d immunized mice respectively. The square regions in white dashed line are cropped images from full western blot images (magnification 100x field of view, Olympus IX73/DP72, cellSens standard software)

Supplementary Table S1~S5

Supplementary Table S1. Significant alignment by computer analysis for searching sequence homology to HCMVpp65_428-437._

| Description | Score | Expect | Identities | Positives | Features |
| --- | --- | --- | --- | --- | --- |
| Transcription initiation factor TFIID subunit 9 | 22.3 | 10 | 7/7 (100%) | 7/7 (100%) |  |
| FERM, ARHGEF and pleckstrin domain containing protein 2 isoforms | 21,8 | 14 | 7/8 (88%) | 7/8 (87%) |  |
| ADP-ribosylation factor-like protein 6-interacting protein 4 isoforms | 21  12.1 | 29  64408 | 7/8 (100%)  4/7(57%) | 7/8 (87)  4/7 (57%) |    |
| WASH complex subunit 5 isoforms | 20.2 | 59 | 6/7 (86%) | 6/7 (85%) |  |
| 60S ribosomal protein | 20.2 | 59 | 6/6 (100%) | 6/6 (100%) |  |

| Nuclear pattern | SA-C3d  (n= 5) | TAF9_134-144_  (n=10) | HCMVpp65_422-439_  (n=10) |
| --- | --- | --- | --- |
| Centriole | 0 | 2 | 5 |
| MSA II | 0 | 3 | 5 |
| Nuclear dots | 0 | 2 | 4 |
| Nuclear rim | 0 | 2 | 2 |
| Nucleosome/chromatin | 0 | 3 | 5 |
| Speckled pattern | 0 | 3 | 5 |

Supplementary Table S2. Anti-nuclear antibody staining of sera from BALB/c mice that received SA-C3d, TAF9_134-144_, and HCMVpp65_422-439_ at 12 weeks after immunization

MSA II: mitotic spindle type II

| Antibody isotypes | SA-C3d  n=5 | TAF9_134-144_  n=10 | HCMVpp65_422-439_  n=10 |
| --- | --- | --- | --- |
| IgG+IgM | 0 | 1 | 6 |
| IgG alone | 0 | 3 | 2 |
| IgM alone | 1 | 1 | 1 |
| All negative | 4 | 5 | 1 |
| IgG subclasses | n=0 | n=4 | n=8 |
| IgG_1_+IgG_2a_+IgG_3_ | 0 | 2 | 1 |
| IgG_1_+IgG_2b_+IgG_3_ | 0 | 2 | 1 |
| IgG_1_+IgG_3_ | 0 | 0 | 6 |
| IgG_1_ | 0 | 0 | 0 |

Supplementary Table S3. Summary of anti-dsDNA activities in immunized mice.

Supplementary Table S4. Summary of the antibody isotypes deposited in glomeruli.

| No. of weeks  post-immunization | SA-C3d  n=5 | HCMVpp65_422-439_  n=10 | TAF9_134-144_  n=10 |
| --- | --- | --- | --- |
| 4 | 1/5, 0/5, 0/5 | 7^(3, 4w)^/10, 7^(2,5w)^/10, 4^(1,3w)^/10 | 6^(2,4w)^/10, 5^(1,4w)^/10, 2^w^/10 |
| 8 | 0/5, 0/5 ,0/5 | 8^(5,3w)^/10, 7^(4.3w)^/10, 7^(3,4w)^/10 | 7^(3,4w)^/10, 7^(2,5w)^/10, 5^(2,3w)^/10 |
| 12 | 0/5, 0/5, 0/5 | 9^(6,3w)^/10, 9^(4,5w)^/10, 8^(4,4w)^/10 | 8^(4,4w)^/10, 7^(4,3w)^/10, 7^(3,4w)^/10 |
| 14 | 0/5, 0/5 ,0/5 | 7^(5,2w)^/10, 7^(3.4w)^/10, 6^(2,4w)^/10 | 5^(2,3w)^/10, 4^(2,2w)^/10, 4^(1,3w)^/10 |
| 16 | 0/5, 0/5, 0/5 | 6^(3,3w)^/10, 5^(2,3w)^/10, 4^(2.2w)^/10 | 4^(1,3w)^/10, 3^(1,2w)^/10, 2^(1,1w)^/10 |

Mice sera were used at dilution of 1:20, 1:40 or 1:80. w: weak response.

Supplementary Table S5. Summary of anti-dsDNA antibody response, proteinuria and IgG deposition in HCMVpp65_422-439_, TAF9_134-144_ and SA-C3d immunized mice.

|  | *C. luciliae* stain | | Proteinuria levels | | | Ig deposition | IgG subclass | |  |
| --- | --- | --- | --- | --- | --- | --- | --- | --- | --- |
| No. of weeks  after immunization | 12 | 16 | 12 | 16 | 16 | | | 16 | |
| SA-C3d group (n=5) | | | | | | | | | |
| uncut | - | - | - | - | - | | |  | |
| L1 | - | - | + | + | IgM | | |  | |
| R1 | - | - | - | - | - | | |  | |
| L1R1 | - | - | - | - | - | | |  | |
| R2 | - | - | - | - | - | | |  | |
| TAF9_134-144_ groups (n=10) | | | | | | | | | |
| Group1-uncut | W | - | + | - |  | | |  | |
| Group1--L1 | W | - | + | - | IgG+IgM | | | IgG1/IgG2a/IgG3 | |
| Group1--R1 | + | + | ++ | ++ | IgG | | | IgG1/IgG2b/IgG3 | |
| Group1--L1R1 | - | - | - | - | - | | |  | |
| Group1--R2 | - | - | - | - | - | | |  | |
| Group2-uncut | - | - | - | + | IgM | | |  | |
| Group2-L1 | - | - | + | - | - | | |  | |
| Group2-R1 | + | W | + | + | IgG | | | IgG1/IgG2b/IgG3 | |
| Group2-L1R1 | - | - | - | - | - | | |  | |
| Group2-R2 | W | - | - | + | IgG | | | IgG1/IgG2a/IgG3 | |
| HCMVpp65_422-439_ groups (n=10) | | | | | | | | | |
| Group1-uncut | - | - | - | - |  | | |  | |
| Group1-L1 | - | - | - | + | IgM | | |  | |
| Group1-R1 | W | - | +++ | ++ | IgG+IgM | | | IgG1/IgG3 | |
| Group1-L1R1 | + | W | +++ | +++ | IgG+IgM | | | IgG1/IgG3 | |
| Group1-R2 | W | - | ++ | ++ | IgG | | | IgG1/IgG2a/IgG3 | |
| Group 2-uncut | - | - | - | - | IgG | | | IgG1/IgG3 | |
| Group 2-L1 | W | - | + | + | IgG/IgM | | | IgG1/IgG3 | |
| Group 2-R1 | + | + | ++ | +++ | IgG+IgM | | | IgG1/IgG3 | |
| Group 2-L1R1 | W | W | + | ++ | IgG+IgM | | | IgG1/IgG2b/IgG3 | |
| Group 2-R2 | + | + | ++ | +++ | IgG+IgM | | | IgG1/IgG3 | |

*C. luciliae* immunofluorescence staining was used for detection of anti-dsDNA activity, +: positive, W: weak response and -: negative. Proteinuria level was measured by proteinuria strip. -: negative, +: >30 mg/dL, ++: >100 mg/dL, +++: >500 mg/dL. Ear holes produced by an ear punch device is used to identify individual mouse. L: left ear; R: right ear.
